# 3,3′-Dibenzyl-2,2′-dimethyl-1,1′-methyl­enediimidazolium dipicrate

**DOI:** 10.1107/S1600536808006272

**Published:** 2008-03-12

**Authors:** Chuan-Ming Jin, Ling-Yan Wu, Xiao-Xia Lu, Jing-Jing Hu

**Affiliations:** a Hubei Key Laboratory of Bioanalytic Techniques, Department of Chemistry and Environmental Engineering, Hubei Normal University, Huangshi 435002, People’s Republic of China

## Abstract

In the title salt, C_23_H_26_N_4_^2+^·2C_6_H_2_N_3_O_7_^−^, the dihedral angle between the imidazolium rings in the dication is 69.9 (1)°. The aromatic ring of the benzyl group is almost perpendicular to the *N*-heterocyclic ring that is directly connected to it [dihedral angles = 83.2 (2) and 77.3 (3)°].

## Related literature

For the synthesis, see: Jin *et al.* (2005 ▶). For background literature on ‘green chemistry’, see: Singh *et al.* (2006 ▶). For background literature on energetic ionic salts, see: Wang *et al.* (2007 ▶).
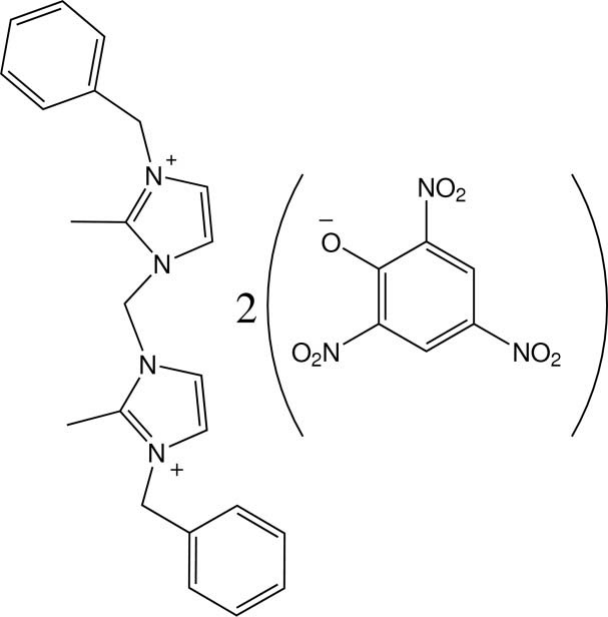


## Experimental

### 

#### Crystal data

C_23_H_26_N_4_^2+^·2C_6_H_2_N_3_O_7_^−^
*M* *_r_* = 814.69
Triclinic, 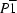

*a* = 12.2842 (8) Å
*b* = 12.6802 (8) Å
*c* = 12.9175 (8) Å
α = 65.691 (1)°
β = 77.601 (1)°
γ = 80.003 (1)°
*V* = 1782.7 (2) Å^3^
*Z* = 2
Mo *K*α radiation
μ = 0.12 mm^−1^
*T* = 294 (2) K
0.30 × 0.20 × 0.13 mm

#### Data collection

Bruker SMART APEX CCD area-detector diffractometer
Absorption correction: none
11547 measured reflections
6921 independent reflections
4320 reflections with *I* > 2σ(*I*)
*R*_int_ = 0.042

#### Refinement

*R*[*F* ^2^ > 2σ(*F* ^2^)] = 0.052
*wR*(*F* ^2^) = 0.130
*S* = 0.93
6921 reflections
534 parameters
H-atom parameters constrained
Δρ_max_ = 0.35 e Å^−3^
Δρ_min_ = −0.21 e Å^−3^

### 

Data collection: *SMART*, (Bruker, 2001 ▶); cell refinement: *SAINT-Plus* (Bruker, 2001 ▶); data reduction: *SAINT-Plus*; program(s) used to solve structure: *SHELXS97* (Sheldrick, 2008 ▶); program(s) used to refine structure: *SHELXL97* (Sheldrick, 2008 ▶); molecular graphics: *SHELXTL* (Sheldrick, 2008 ▶); software used to prepare material for publication: *SHELXTL*.

## Supplementary Material

Crystal structure: contains datablocks I, global. DOI: 10.1107/S1600536808006272/ng2431sup1.cif

Structure factors: contains datablocks I. DOI: 10.1107/S1600536808006272/ng2431Isup2.hkl

Additional supplementary materials:  crystallographic information; 3D view; checkCIF report

## Figures and Tables

**Fig. 1.**
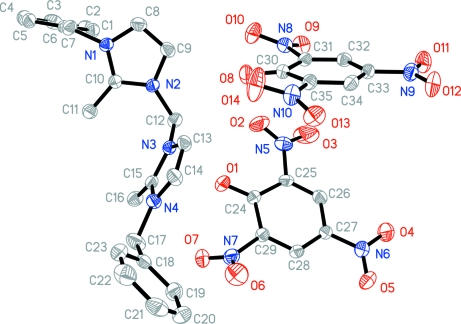
The structure of (I) showing the atom-numbering scheme. Displacement ellipsoids are drawn at the 30% probability level and H atoms have been omitted.

## supplementary crystallographic information

### Comment

Polynitrogen heterocyclic organic salts with low melting points are a new class of energetic materials that has attracted considerable interest because of their "green chemistry" properties (Singh *et al.*, 2006). Picric acid is a polynitrogen compound with explosive character. Imidazolium-based or triazolium-based dication picrate salts are good candidates for energetic ionic salts (Wang *et al.*, 2007). Based on this, the title organic salt (scheme 1) was therefore prepared and its structure is reported here.

The asymmetric unit of the title compound contains one 1, 1'-Methylenebis (2-methyl-3-benzyl- imidazolium) dication and two picrate anions (Figure 1). The dihedral angle between the imidazolium rings in the dication is 69.9 (1)°. The benzene ring of benzenyl group is almost perpendicular with the imidazole ring which is directly connected with them, making the dihedral angle of 96.8 (2)° and 102.7 (3)°, respectively. And the dihedral angle between the benzene ring of the two independent picrate anions is 42.8 (2)°. The molecules were packed by the weak C—H···O interaction between cations and anions (Table 1).

### Experimental

The title salt (C_23_H_26_N_4_)^2+^.2(C_6_H_2_N_3_O_7_)^-^ was synthesized using a slightly modified literature method (Jin *et al.*, 2005). It was crystallized by slow evaporation of an acetonitrile solution of the salt.

### Refinement

H atoms were positioned geometrically with C—H bond lengths fixed to 0.93 (aromatic CH),0.97 (methylene CH_2_) or 0.96Å (methyl CH_3_). A riding model was used during the refinement process. The *U*_iso_ parameters for H atoms were constrained to be 1.2*U*_eq_ of the carrier C atom for aromatic and methylene groups, and 1.5*U*_eq_ of the carrier C atom for methyl groups.

### Crystal data

**Table:**

C_23_H_26_N_4_^2+^·2C_6_H_2_N_3_O_7_^–^	*Z* = 2
*M**_r_* = 814.69	*F*_000_ = 844
Triclinic, *P*1	*D*_x_ = 1.518 Mg m^−^^3^
*a* = 12.2842 (8) Å	Mo *K*α radiation λ = 0.71073 Å
*b* = 12.6802 (8) Å	Cell parameters from 2808 reflections
*c* = 12.9175 (8) Å	θ = 2.2–23.4º
α = 65.691 (1)º	µ = 0.12 mm^−^^1^
β = 77.601 (1)º	*T* = 294 (2) K
γ = 80.003 (1)º	Block, yellow
*V* = 1782.7 (2) Å^3^	0.30 × 0.20 × 0.13 mm

### Data collection

**Table:**

Bruker SMART APEX CCD area-detector diffractometer	4320 reflections with *I* > 2σ(*I*)
Radiation source: fine-focus sealed tube	*R*_int_ = 0.042
Monochromator: graphite	θ_max_ = 26.0º
*T* = 294(2) K	θ_min_ = 1.7º
φ and ω scans	*h* = −15→13
Absorption correction: none	*k* = −15→15
11547 measured reflections	*l* = −15→13
6921 independent reflections	

### Refinement

**Table:**

Refinement on *F*^2^	Secondary atom site location: difference Fourier map
Least-squares matrix: full	Hydrogen site location: inferred from neighbouring sites
*R*[*F*^2^ > 2σ(*F*^2^)] = 0.052	H-atom parameters constrained
*wR*(*F*^2^) = 0.130	*w* = 1/[σ^2^(*F*_o_^2^) + (0.0604*P*)^2^] where *P* = (*F*_o_^2^ + 2*F*_c_^2^)/3
*S* = 0.93	(Δ/σ)_max_ = 0.002
6921 reflections	Δρ_max_ = 0.35 e Å^−^^3^
534 parameters	Δρ_min_ = −0.21 e Å^−^^3^
Primary atom site location: structure-invariant direct methods	Extinction correction: none

### Special details

**Table:**

Geometry. All e.s.d.'s (except the e.s.d. in the dihedral angle between two l.s. planes) are estimated using the full covariance matrix. The cell e.s.d.'s are taken into account individually in the estimation of e.s.d.'s in distances, angles and torsion angles; correlations between e.s.d.'s in cell parameters are only used when they are defined by crystal symmetry. An approximate (isotropic) treatment of cell e.s.d.'s is used for estimating e.s.d.'s involving l.s. planes.
Refinement. Refinement of *F*^2^ against ALL reflections. The weighted *R*-factor *wR* and goodness of fit *S* are based on *F*^2^, conventional *R*-factors *R* are based on *F*, with *F* set to zero for negative *F*^2^. The threshold expression of *F*^2^ > σ(*F*^2^) is used only for calculating *R*-factors(gt) *etc*. and is not relevant to the choice of reflections for refinement. *R*-factors based on *F*^2^ are statistically about twice as large as those based on *F*, and *R*- factors based on ALL data will be even larger.

### Fractional atomic coordinates and isotropic or equivalent isotropic displacement parameters (Å^2^)

**Table:**

	*x*	*y*	*z*	*U*_iso_*/*U*_eq_	
C1	−0.32013 (19)	0.2269 (2)	0.94028 (19)	0.0481 (6)	
H1	−0.2439	0.2078	0.9222	0.058*	
C2	−0.3562 (2)	0.2944 (2)	1.0040 (2)	0.0589 (7)	
H2	−0.3043	0.3206	1.0286	0.071*	
C3	−0.4689 (3)	0.3231 (2)	1.0312 (2)	0.0670 (8)	
H3	−0.4934	0.3679	1.0749	0.080*	
C4	−0.5445 (2)	0.2857 (2)	0.9939 (2)	0.0655 (8)	
H4	−0.6206	0.3059	1.0116	0.079*	
C5	−0.50921 (19)	0.2180 (2)	0.9300 (2)	0.0525 (6)	
H5	−0.5615	0.1928	0.9050	0.063*	
C6	−0.39585 (18)	0.18755 (19)	0.90311 (18)	0.0415 (5)	
C7	−0.36084 (17)	0.1092 (2)	0.8373 (2)	0.0462 (6)	
H7A	−0.3766	0.0305	0.8887	0.055*	
H7B	−0.4053	0.1343	0.7761	0.055*	
C8	−0.15951 (19)	0.0230 (2)	0.8316 (2)	0.0509 (6)	
H8	−0.1699	−0.0468	0.8947	0.061*	
C9	−0.0615 (2)	0.0584 (2)	0.7674 (2)	0.0500 (6)	
H9	0.0088	0.0179	0.7771	0.060*	
C10	−0.19573 (17)	0.19641 (18)	0.69713 (18)	0.0361 (5)	
C11	−0.25476 (18)	0.30444 (19)	0.6275 (2)	0.0496 (6)	
H11A	−0.2522	0.3055	0.5523	0.074*	
H11B	−0.2196	0.3694	0.6209	0.074*	
H11C	−0.3313	0.3095	0.6636	0.074*	
C12	0.00031 (18)	0.2391 (2)	0.59965 (18)	0.0460 (6)	
H12A	−0.0163	0.3177	0.5971	0.055*	
H12B	0.0728	0.2092	0.6240	0.055*	
C13	0.04891 (17)	0.1502 (2)	0.4523 (2)	0.0459 (6)	
H13	0.0757	0.0765	0.4995	0.055*	
C14	0.04486 (18)	0.1872 (2)	0.3408 (2)	0.0504 (6)	
H14	0.0683	0.1438	0.2956	0.060*	
C15	−0.02355 (16)	0.33463 (19)	0.39257 (19)	0.0394 (5)	
C16	−0.06630 (19)	0.45312 (19)	0.3845 (2)	0.0519 (6)	
H16A	−0.0138	0.5066	0.3319	0.078*	
H16B	−0.0760	0.4560	0.4590	0.078*	
H16C	−0.1369	0.4740	0.3572	0.078*	
C17	−0.0239 (2)	0.3813 (2)	0.1872 (2)	0.0665 (8)	
H17A	0.0127	0.4514	0.1629	0.080*	
H17B	−0.1039	0.4033	0.1911	0.080*	
C18	0.01479 (19)	0.3287 (2)	0.09860 (19)	0.0451 (6)	
C19	0.1118 (2)	0.3592 (2)	0.0223 (2)	0.0547 (6)	
H19	0.1556	0.4078	0.0297	0.066*	
C20	0.1456 (2)	0.3191 (2)	−0.0650 (2)	0.0618 (7)	
H20	0.2118	0.3404	−0.1159	0.074*	
C21	0.0816 (3)	0.2483 (2)	−0.0764 (2)	0.0656 (8)	
H21	0.1031	0.2223	−0.1362	0.079*	
C22	−0.0139 (3)	0.2153 (2)	−0.0005 (3)	0.0728 (8)	
H22	−0.0564	0.1652	−0.0074	0.087*	
C23	−0.0480 (2)	0.2556 (2)	0.0863 (2)	0.0648 (7)	
H23	−0.1139	0.2333	0.1371	0.078*	
C24	0.27922 (18)	0.40292 (18)	0.42696 (19)	0.0405 (5)	
C25	0.36650 (19)	0.37045 (18)	0.49657 (18)	0.0434 (5)	
C26	0.4775 (2)	0.38252 (19)	0.4525 (2)	0.0496 (6)	
H26	0.5294	0.3626	0.5021	0.059*	
C27	0.51307 (18)	0.42396 (19)	0.3353 (2)	0.0459 (6)	
C28	0.43647 (18)	0.45553 (18)	0.26156 (19)	0.0427 (5)	
H28	0.4601	0.4824	0.1825	0.051*	
C29	0.32617 (18)	0.44706 (19)	0.30531 (19)	0.0415 (5)	
C30	0.27633 (18)	−0.03995 (19)	0.67370 (19)	0.0421 (5)	
C31	0.33425 (18)	−0.02705 (18)	0.75420 (19)	0.0414 (5)	
C32	0.44610 (18)	−0.05648 (18)	0.75761 (19)	0.0435 (5)	
H32	0.4786	−0.0450	0.8104	0.052*	
C33	0.51087 (18)	−0.10330 (18)	0.6825 (2)	0.0445 (6)	
C34	0.46294 (18)	−0.12262 (18)	0.6051 (2)	0.0459 (6)	
H34	0.5069	−0.1543	0.5548	0.055*	
C35	0.35072 (18)	−0.09491 (19)	0.60286 (19)	0.0431 (5)	
N1	−0.24203 (14)	0.10880 (15)	0.78718 (15)	0.0399 (4)	
N2	−0.08471 (14)	0.16696 (15)	0.68376 (14)	0.0389 (4)	
N3	0.00576 (13)	0.24197 (15)	0.48479 (15)	0.0390 (4)	
N4	−0.00050 (14)	0.30187 (16)	0.30376 (15)	0.0448 (5)	
N5	0.3367 (2)	0.3203 (2)	0.6210 (2)	0.0684 (6)	
N6	0.63001 (18)	0.4290 (2)	0.2899 (2)	0.0686 (6)	
N7	0.24869 (19)	0.4833 (2)	0.22345 (19)	0.0621 (6)	
N8	0.27178 (18)	0.01525 (17)	0.84116 (17)	0.0500 (5)	
N9	0.62976 (17)	−0.13317 (18)	0.6852 (2)	0.0575 (6)	
N10	0.30666 (18)	−0.11954 (18)	0.5197 (2)	0.0577 (6)	
O1	0.17779 (13)	0.39644 (14)	0.46220 (14)	0.0561 (4)	
O2	0.24912 (19)	0.2763 (2)	0.66457 (17)	0.1098 (9)	
O3	0.4034 (2)	0.3208 (2)	0.67838 (18)	0.1245 (10)	
O4	0.69643 (16)	0.4028 (2)	0.3567 (2)	0.1142 (9)	
O5	0.65990 (15)	0.4578 (2)	0.18593 (19)	0.0872 (7)	
O6	0.27181 (19)	0.4481 (2)	0.14539 (19)	0.0982 (7)	
O7	0.16644 (16)	0.5492 (2)	0.23414 (17)	0.0850 (7)	
O8	0.17702 (13)	−0.00456 (15)	0.66426 (14)	0.0600 (5)	
O9	0.32355 (15)	0.05338 (15)	0.88774 (16)	0.0654 (5)	
O10	0.17139 (15)	0.00864 (17)	0.86778 (16)	0.0724 (5)	
O11	0.67036 (14)	−0.11617 (17)	0.75553 (18)	0.0748 (6)	
O12	0.68460 (14)	−0.17238 (18)	0.61513 (19)	0.0798 (6)	
O13	0.37262 (15)	−0.13424 (16)	0.43990 (15)	0.0676 (5)	
O14	0.20723 (16)	−0.1274 (2)	0.5327 (2)	0.1081 (9)	

### Atomic displacement parameters (Å^2^)

**Table:**

	*U*^11^	*U*^22^	*U*^33^	*U*^12^	*U*^13^	*U*^23^
C1	0.0441 (13)	0.0571 (15)	0.0434 (14)	−0.0051 (11)	−0.0059 (11)	−0.0202 (12)
C2	0.0766 (19)	0.0591 (17)	0.0463 (15)	−0.0073 (14)	−0.0163 (14)	−0.0222 (13)
C3	0.088 (2)	0.0614 (18)	0.0456 (16)	0.0101 (16)	−0.0061 (15)	−0.0241 (14)
C4	0.0575 (17)	0.0731 (19)	0.0527 (17)	0.0128 (14)	−0.0009 (14)	−0.0226 (15)
C5	0.0418 (13)	0.0667 (17)	0.0431 (14)	−0.0019 (12)	−0.0034 (11)	−0.0185 (13)
C6	0.0403 (13)	0.0490 (14)	0.0311 (12)	−0.0053 (10)	−0.0017 (10)	−0.0132 (11)
C7	0.0407 (13)	0.0592 (15)	0.0404 (13)	−0.0115 (11)	0.0005 (10)	−0.0220 (12)
C8	0.0550 (15)	0.0477 (15)	0.0393 (14)	0.0017 (12)	−0.0031 (12)	−0.0112 (11)
C9	0.0480 (14)	0.0529 (15)	0.0416 (14)	0.0096 (11)	−0.0077 (12)	−0.0164 (12)
C10	0.0388 (12)	0.0409 (13)	0.0325 (12)	−0.0010 (10)	−0.0034 (10)	−0.0203 (10)
C11	0.0484 (14)	0.0507 (15)	0.0458 (14)	−0.0009 (11)	−0.0047 (11)	−0.0177 (12)
C12	0.0432 (13)	0.0617 (15)	0.0381 (13)	−0.0116 (11)	−0.0030 (11)	−0.0235 (12)
C13	0.0404 (13)	0.0507 (14)	0.0446 (14)	0.0066 (11)	−0.0074 (11)	−0.0204 (12)
C14	0.0492 (14)	0.0603 (16)	0.0438 (15)	0.0133 (12)	−0.0101 (11)	−0.0284 (13)
C15	0.0314 (11)	0.0468 (14)	0.0379 (13)	−0.0058 (10)	0.0016 (10)	−0.0171 (11)
C16	0.0515 (14)	0.0507 (15)	0.0504 (15)	−0.0057 (11)	0.0008 (12)	−0.0207 (12)
C17	0.0777 (19)	0.0726 (18)	0.0427 (15)	0.0220 (14)	−0.0173 (14)	−0.0236 (14)
C18	0.0444 (14)	0.0523 (14)	0.0332 (13)	0.0045 (11)	−0.0125 (11)	−0.0118 (11)
C19	0.0511 (15)	0.0636 (17)	0.0554 (16)	−0.0110 (12)	−0.0115 (13)	−0.0253 (14)
C20	0.0557 (16)	0.0744 (19)	0.0478 (16)	−0.0050 (14)	0.0026 (13)	−0.0221 (14)
C21	0.091 (2)	0.0614 (18)	0.0455 (16)	0.0103 (16)	−0.0204 (16)	−0.0236 (14)
C22	0.089 (2)	0.069 (2)	0.072 (2)	−0.0222 (17)	−0.0330 (19)	−0.0247 (17)
C23	0.0486 (15)	0.081 (2)	0.0530 (17)	−0.0187 (14)	−0.0067 (13)	−0.0101 (15)
C24	0.0424 (13)	0.0368 (12)	0.0429 (14)	−0.0052 (10)	0.0003 (11)	−0.0191 (11)
C25	0.0537 (15)	0.0397 (13)	0.0314 (12)	−0.0039 (11)	−0.0046 (11)	−0.0098 (10)
C26	0.0508 (15)	0.0493 (15)	0.0490 (15)	−0.0037 (11)	−0.0170 (12)	−0.0156 (12)
C27	0.0378 (13)	0.0464 (14)	0.0479 (15)	−0.0074 (10)	−0.0067 (11)	−0.0116 (12)
C28	0.0451 (13)	0.0460 (13)	0.0367 (13)	−0.0089 (10)	−0.0024 (11)	−0.0159 (11)
C29	0.0407 (13)	0.0477 (13)	0.0409 (13)	−0.0071 (10)	−0.0094 (11)	−0.0196 (11)
C30	0.0370 (13)	0.0434 (13)	0.0413 (13)	−0.0025 (10)	−0.0048 (10)	−0.0132 (11)
C31	0.0405 (13)	0.0413 (13)	0.0390 (13)	−0.0033 (10)	−0.0017 (10)	−0.0148 (11)
C32	0.0466 (14)	0.0401 (13)	0.0437 (13)	−0.0088 (10)	−0.0085 (11)	−0.0138 (11)
C33	0.0352 (12)	0.0419 (13)	0.0521 (15)	−0.0059 (10)	−0.0057 (11)	−0.0138 (12)
C34	0.0423 (13)	0.0418 (13)	0.0515 (15)	−0.0075 (10)	0.0039 (11)	−0.0201 (12)
C35	0.0407 (13)	0.0460 (14)	0.0462 (14)	−0.0083 (10)	−0.0062 (11)	−0.0202 (11)
N1	0.0393 (10)	0.0459 (11)	0.0335 (10)	−0.0048 (9)	0.0006 (8)	−0.0173 (9)
N2	0.0367 (10)	0.0467 (11)	0.0313 (10)	−0.0036 (8)	−0.0005 (8)	−0.0158 (9)
N3	0.0349 (10)	0.0497 (11)	0.0341 (10)	−0.0057 (8)	−0.0040 (8)	−0.0181 (9)
N4	0.0437 (11)	0.0553 (12)	0.0326 (10)	0.0057 (9)	−0.0050 (9)	−0.0189 (9)
N5	0.0785 (17)	0.0743 (16)	0.0429 (14)	−0.0068 (13)	−0.0128 (13)	−0.0121 (12)
N6	0.0458 (14)	0.0775 (16)	0.0673 (17)	−0.0099 (12)	−0.0095 (13)	−0.0115 (13)
N7	0.0525 (14)	0.0846 (17)	0.0495 (14)	−0.0234 (13)	−0.0088 (11)	−0.0195 (13)
N8	0.0543 (13)	0.0482 (12)	0.0478 (13)	−0.0033 (10)	−0.0080 (11)	−0.0196 (10)
N9	0.0422 (12)	0.0566 (13)	0.0734 (16)	−0.0063 (10)	−0.0057 (12)	−0.0262 (12)
N10	0.0500 (13)	0.0636 (14)	0.0708 (15)	−0.0052 (11)	−0.0086 (12)	−0.0380 (12)
O1	0.0449 (10)	0.0662 (11)	0.0544 (11)	−0.0104 (8)	0.0050 (8)	−0.0249 (9)
O2	0.0825 (15)	0.161 (2)	0.0478 (13)	−0.0346 (16)	0.0045 (12)	−0.0007 (14)
O3	0.149 (2)	0.174 (3)	0.0492 (14)	−0.059 (2)	−0.0279 (15)	−0.0196 (15)
O4	0.0495 (12)	0.171 (2)	0.0856 (16)	−0.0216 (14)	−0.0277 (12)	−0.0015 (16)
O5	0.0522 (12)	0.1214 (18)	0.0681 (14)	−0.0155 (11)	0.0084 (11)	−0.0232 (13)
O6	0.1076 (17)	0.146 (2)	0.0743 (15)	−0.0297 (15)	−0.0295 (13)	−0.0616 (15)
O7	0.0453 (11)	0.1186 (18)	0.0711 (14)	−0.0012 (11)	−0.0164 (10)	−0.0164 (13)
O8	0.0402 (10)	0.0855 (13)	0.0561 (11)	0.0074 (9)	−0.0116 (8)	−0.0328 (10)
O9	0.0721 (12)	0.0719 (12)	0.0671 (12)	−0.0046 (9)	−0.0167 (10)	−0.0399 (10)
O10	0.0501 (11)	0.1032 (15)	0.0756 (13)	−0.0103 (10)	0.0066 (10)	−0.0534 (12)
O11	0.0498 (11)	0.0979 (15)	0.0884 (15)	−0.0044 (10)	−0.0233 (10)	−0.0429 (12)
O12	0.0420 (10)	0.1008 (15)	0.1119 (17)	0.0027 (10)	−0.0036 (11)	−0.0643 (14)
O13	0.0761 (13)	0.0797 (13)	0.0572 (12)	−0.0133 (10)	−0.0044 (10)	−0.0375 (10)
O14	0.0500 (12)	0.180 (2)	0.157 (2)	−0.0119 (13)	−0.0157 (13)	−0.127 (2)

### Geometric parameters (Å, °)

**Table:**

C1—C6	1.377 (3)	C18—C23	1.380 (3)
C1—C2	1.378 (3)	C19—C20	1.379 (3)
C1—H1	0.9300	C19—H19	0.9300
C2—C3	1.377 (4)	C20—C21	1.361 (4)
C2—H2	0.9300	C20—H20	0.9300
C3—C4	1.360 (4)	C21—C22	1.360 (4)
C3—H3	0.9300	C21—H21	0.9300
C4—C5	1.381 (3)	C22—C23	1.374 (4)
C4—H4	0.9300	C22—H22	0.9300
C5—C6	1.387 (3)	C23—H23	0.9300
C5—H5	0.9300	C24—O1	1.236 (2)
C6—C7	1.512 (3)	C24—C25	1.443 (3)
C7—N1	1.464 (3)	C24—C29	1.454 (3)
C7—H7A	0.9700	C25—C26	1.369 (3)
C7—H7B	0.9700	C25—N5	1.451 (3)
C8—C9	1.339 (3)	C26—C27	1.378 (3)
C8—N1	1.376 (3)	C26—H26	0.9300
C8—H8	0.9300	C27—C28	1.376 (3)
C9—N2	1.384 (3)	C27—N6	1.432 (3)
C9—H9	0.9300	C28—C29	1.356 (3)
C10—N1	1.335 (3)	C28—H28	0.9300
C10—N2	1.343 (2)	C29—N7	1.455 (3)
C10—C11	1.460 (3)	C30—O8	1.238 (2)
C11—H11A	0.9600	C30—C35	1.450 (3)
C11—H11B	0.9600	C30—C31	1.453 (3)
C11—H11C	0.9600	C31—C32	1.365 (3)
C12—N3	1.457 (3)	C31—N8	1.457 (3)
C12—N2	1.461 (3)	C32—C33	1.380 (3)
C12—H12A	0.9700	C32—H32	0.9300
C12—H12B	0.9700	C33—C34	1.383 (3)
C13—C14	1.328 (3)	C33—N9	1.448 (3)
C13—N3	1.380 (3)	C34—C35	1.366 (3)
C13—H13	0.9300	C34—H34	0.9300
C14—N4	1.383 (3)	C35—N10	1.459 (3)
C14—H14	0.9300	N5—O2	1.215 (3)
C15—N4	1.334 (3)	N5—O3	1.219 (3)
C15—N3	1.347 (3)	N6—O4	1.222 (3)
C15—C16	1.471 (3)	N6—O5	1.226 (3)
C16—H16A	0.9600	N7—O7	1.217 (3)
C16—H16B	0.9600	N7—O6	1.225 (3)
C16—H16C	0.9600	N8—O10	1.215 (2)
C17—N4	1.483 (3)	N8—O9	1.229 (2)
C17—C18	1.505 (3)	N9—O11	1.230 (3)
C17—H17A	0.9700	N9—O12	1.230 (3)
C17—H17B	0.9700	N10—O14	1.213 (2)
C18—C19	1.372 (3)	N10—O13	1.225 (2)
			
C6—C1—C2	120.7 (2)	C20—C21—H21	120.0
C6—C1—H1	119.7	C21—C22—C23	120.4 (3)
C2—C1—H1	119.7	C21—C22—H22	119.8
C3—C2—C1	120.0 (2)	C23—C22—H22	119.8
C3—C2—H2	120.0	C22—C23—C18	120.6 (2)
C1—C2—H2	120.0	C22—C23—H23	119.7
C4—C3—C2	119.9 (2)	C18—C23—H23	119.7
C4—C3—H3	120.1	O1—C24—C25	126.5 (2)
C2—C3—H3	120.1	O1—C24—C29	122.8 (2)
C3—C4—C5	120.6 (2)	C25—C24—C29	110.62 (19)
C3—C4—H4	119.7	C26—C25—C24	124.0 (2)
C5—C4—H4	119.7	C26—C25—N5	117.1 (2)
C4—C5—C6	120.1 (2)	C24—C25—N5	118.9 (2)
C4—C5—H5	120.0	C25—C26—C27	120.5 (2)
C6—C5—H5	120.0	C25—C26—H26	119.7
C1—C6—C5	118.8 (2)	C27—C26—H26	119.7
C1—C6—C7	122.70 (19)	C28—C27—C26	120.0 (2)
C5—C6—C7	118.5 (2)	C28—C27—N6	119.6 (2)
N1—C7—C6	113.15 (17)	C26—C27—N6	120.3 (2)
N1—C7—H7A	108.9	C29—C28—C27	119.4 (2)
C6—C7—H7A	108.9	C29—C28—H28	120.3
N1—C7—H7B	108.9	C27—C28—H28	120.3
C6—C7—H7B	108.9	C28—C29—C24	125.5 (2)
H7A—C7—H7B	107.8	C28—C29—N7	117.1 (2)
C9—C8—N1	107.5 (2)	C24—C29—N7	117.39 (19)
C9—C8—H8	126.3	O8—C30—C35	124.4 (2)
N1—C8—H8	126.3	O8—C30—C31	124.0 (2)
C8—C9—N2	106.9 (2)	C35—C30—C31	111.46 (18)
C8—C9—H9	126.6	C32—C31—C30	124.1 (2)
N2—C9—H9	126.6	C32—C31—N8	115.8 (2)
N1—C10—N2	107.06 (18)	C30—C31—N8	120.05 (19)
N1—C10—C11	126.33 (19)	C31—C32—C33	119.9 (2)
N2—C10—C11	126.6 (2)	C31—C32—H32	120.1
C10—C11—H11A	109.5	C33—C32—H32	120.1
C10—C11—H11B	109.5	C32—C33—C34	120.5 (2)
H11A—C11—H11B	109.5	C32—C33—N9	119.9 (2)
C10—C11—H11C	109.5	C34—C33—N9	119.7 (2)
H11A—C11—H11C	109.5	C35—C34—C33	119.7 (2)
H11B—C11—H11C	109.5	C35—C34—H34	120.2
N3—C12—N2	112.33 (17)	C33—C34—H34	120.2
N3—C12—H12A	109.1	C34—C35—C30	124.2 (2)
N2—C12—H12A	109.1	C34—C35—N10	116.2 (2)
N3—C12—H12B	109.1	C30—C35—N10	119.54 (19)
N2—C12—H12B	109.1	C10—N1—C8	109.43 (18)
H12A—C12—H12B	107.9	C10—N1—C7	125.02 (18)
C14—C13—N3	107.0 (2)	C8—N1—C7	125.31 (19)
C14—C13—H13	126.5	C10—N2—C9	109.16 (18)
N3—C13—H13	126.5	C10—N2—C12	126.38 (18)
C13—C14—N4	107.88 (19)	C9—N2—C12	124.37 (18)
C13—C14—H14	126.1	C15—N3—C13	109.25 (18)
N4—C14—H14	126.1	C15—N3—C12	126.22 (19)
N4—C15—N3	107.00 (18)	C13—N3—C12	124.42 (19)
N4—C15—C16	124.4 (2)	C15—N4—C14	108.90 (18)
N3—C15—C16	128.5 (2)	C15—N4—C17	122.33 (19)
C15—C16—H16A	109.5	C14—N4—C17	128.77 (19)
C15—C16—H16B	109.5	O2—N5—O3	122.2 (2)
H16A—C16—H16B	109.5	O2—N5—C25	119.8 (2)
C15—C16—H16C	109.5	O3—N5—C25	118.0 (2)
H16A—C16—H16C	109.5	O4—N6—O5	122.5 (2)
H16B—C16—H16C	109.5	O4—N6—C27	118.6 (2)
N4—C17—C18	113.42 (19)	O5—N6—C27	118.9 (2)
N4—C17—H17A	108.9	O7—N7—O6	123.6 (2)
C18—C17—H17A	108.9	O7—N7—C29	118.5 (2)
N4—C17—H17B	108.9	O6—N7—C29	117.9 (2)
C18—C17—H17B	108.9	O10—N8—O9	122.2 (2)
H17A—C17—H17B	107.7	O10—N8—C31	119.3 (2)
C19—C18—C23	118.1 (2)	O9—N8—C31	118.4 (2)
C19—C18—C17	119.8 (2)	O11—N9—O12	123.9 (2)
C23—C18—C17	122.0 (2)	O11—N9—C33	118.5 (2)
C18—C19—C20	121.3 (2)	O12—N9—C33	117.6 (2)
C18—C19—H19	119.3	O14—N10—O13	122.1 (2)
C20—C19—H19	119.3	O14—N10—C35	119.5 (2)
C21—C20—C19	119.5 (2)	O13—N10—C35	118.3 (2)
C21—C20—H20	120.2	C24—O1—H12A	160.3
C19—C20—H20	120.2	N5—O2—O8	126.5 (2)
C22—C21—C20	120.1 (3)	C30—O8—O2	85.51 (14)
C22—C21—H21	120.0		
			
C6—C1—C2—C3	0.0 (4)	C9—C8—N1—C7	−174.9 (2)
C1—C2—C3—C4	−0.7 (4)	C6—C7—N1—C10	−72.2 (3)
C2—C3—C4—C5	0.7 (4)	C6—C7—N1—C8	101.6 (2)
C3—C4—C5—C6	−0.1 (4)	N1—C10—N2—C9	0.1 (2)
C2—C1—C6—C5	0.6 (3)	C11—C10—N2—C9	179.3 (2)
C2—C1—C6—C7	−177.4 (2)	N1—C10—N2—C12	−176.49 (18)
C4—C5—C6—C1	−0.6 (3)	C11—C10—N2—C12	2.7 (3)
C4—C5—C6—C7	177.5 (2)	C8—C9—N2—C10	−0.3 (2)
C1—C6—C7—N1	−17.7 (3)	C8—C9—N2—C12	176.4 (2)
C5—C6—C7—N1	164.3 (2)	N3—C12—N2—C10	−77.6 (3)
N1—C8—C9—N2	0.4 (3)	N3—C12—N2—C9	106.4 (2)
N3—C13—C14—N4	0.1 (3)	N4—C15—N3—C13	0.6 (2)
N4—C17—C18—C19	−102.2 (3)	C16—C15—N3—C13	−175.8 (2)
N4—C17—C18—C23	81.9 (3)	N4—C15—N3—C12	176.95 (17)
C23—C18—C19—C20	0.6 (4)	C16—C15—N3—C12	0.6 (3)
C17—C18—C19—C20	−175.4 (2)	C14—C13—N3—C15	−0.5 (2)
C18—C19—C20—C21	0.2 (4)	C14—C13—N3—C12	−176.88 (19)
C19—C20—C21—C22	−1.4 (4)	N2—C12—N3—C15	112.0 (2)
C20—C21—C22—C23	1.7 (4)	N2—C12—N3—C13	−72.2 (3)
C21—C22—C23—C18	−0.8 (4)	N3—C15—N4—C14	−0.5 (2)
C19—C18—C23—C22	−0.3 (4)	C16—C15—N4—C14	176.1 (2)
C17—C18—C23—C22	175.6 (2)	N3—C15—N4—C17	178.81 (19)
O1—C24—C25—C26	−178.2 (2)	C16—C15—N4—C17	−4.6 (3)
C29—C24—C25—C26	1.3 (3)	C13—C14—N4—C15	0.3 (3)
O1—C24—C25—N5	2.8 (3)	C13—C14—N4—C17	−179.0 (2)
C29—C24—C25—N5	−177.8 (2)	C18—C17—N4—C15	176.6 (2)
C24—C25—C26—C27	−2.4 (4)	C18—C17—N4—C14	−4.2 (4)
N5—C25—C26—C27	176.7 (2)	C26—C25—N5—O2	−158.4 (3)
C25—C26—C27—C28	1.2 (4)	C24—C25—N5—O2	20.7 (4)
C25—C26—C27—N6	−175.8 (2)	C26—C25—N5—O3	19.3 (4)
C26—C27—C28—C29	1.0 (3)	C24—C25—N5—O3	−161.6 (2)
N6—C27—C28—C29	177.9 (2)	C28—C27—N6—O4	178.6 (2)
C27—C28—C29—C24	−2.1 (3)	C26—C27—N6—O4	−4.5 (4)
C27—C28—C29—N7	179.1 (2)	C28—C27—N6—O5	−2.5 (4)
O1—C24—C29—C28	−179.5 (2)	C26—C27—N6—O5	174.5 (2)
C25—C24—C29—C28	1.0 (3)	C28—C29—N7—O7	−131.0 (2)
O1—C24—C29—N7	−0.7 (3)	C24—C29—N7—O7	50.1 (3)
C25—C24—C29—N7	179.78 (19)	C28—C29—N7—O6	47.0 (3)
O8—C30—C31—C32	−173.8 (2)	C24—C29—N7—O6	−131.9 (2)
C35—C30—C31—C32	3.8 (3)	C32—C31—N8—O10	−157.1 (2)
O8—C30—C31—N8	8.7 (3)	C30—C31—N8—O10	20.6 (3)
C35—C30—C31—N8	−173.68 (18)	C32—C31—N8—O9	20.7 (3)
C30—C31—C32—C33	−1.0 (3)	C30—C31—N8—O9	−161.6 (2)
N8—C31—C32—C33	176.51 (19)	C32—C33—N9—O11	0.2 (3)
C31—C32—C33—C34	−1.2 (3)	C34—C33—N9—O11	−179.4 (2)
C31—C32—C33—N9	179.24 (19)	C32—C33—N9—O12	−178.5 (2)
C32—C33—C34—C35	0.1 (3)	C34—C33—N9—O12	1.9 (3)
N9—C33—C34—C35	179.7 (2)	C34—C35—N10—O14	160.1 (2)
C33—C34—C35—C30	3.2 (3)	C30—C35—N10—O14	−22.1 (3)
C33—C34—C35—N10	−179.2 (2)	C34—C35—N10—O13	−18.2 (3)
O8—C30—C35—C34	172.7 (2)	C30—C35—N10—O13	159.6 (2)
C31—C30—C35—C34	−4.8 (3)	C25—C24—O1—H12A	−34.2
O8—C30—C35—N10	−4.8 (3)	C29—C24—O1—H12A	146.4
C31—C30—C35—N10	177.59 (19)	O3—N5—O2—O8	−108.7 (3)
N2—C10—N1—C8	0.2 (2)	C25—N5—O2—O8	69.0 (3)
C11—C10—N1—C8	−179.0 (2)	C35—C30—O8—O2	−130.9 (2)
N2—C10—N1—C7	174.79 (18)	C31—C30—O8—O2	46.4 (2)
C11—C10—N1—C7	−4.4 (3)	N5—O2—O8—C30	36.4 (2)
C9—C8—N1—C10	−0.3 (3)		

### Hydrogen-bond geometry (Å, °)

**Table:**

*D*—H···*A*	*D*—H	H···*A*	*D*···*A*	*D*—H···*A*
C19—H19···O6	0.93	2.52	3.354 (3)	150
C16—H16A···O7	0.96	2.33	3.210 (3)	152
C13—H13···O8	0.93	2.45	3.190 (3)	136
C9—H9···O10	0.93	2.49	3.249 (3)	139
C9—H9···O8	0.93	2.31	3.063 (3)	138
C16—H16C···O4^i^	0.96	2.38	3.211 (3)	145
C11—H11A···O4^i^	0.96	2.48	3.347 (3)	150
C5—H5···O9^i^	0.93	2.55	3.433 (3)	159
C16—H16B···O1^ii^	0.96	2.51	3.237 (3)	132

Symmetry codes: (i) *x*−1, *y*, *z*; (ii) −*x*, −*y*+1, −*z*+1.
